# Supplementary material for: Unraveling the physiological responses of morphologically distinct corals to low oxygen
Source: PeerJ. 2024 Sep 23;12:e18095. doi: 10.7717/peerj.18095 (PMC11426318; doi:10.7717/peerj.18095)

Table S1. Environmental parameters measured by AAQ-RINKO *in situ* (mean ± SE, n = 3)*.*

|  |  |  |  |  |  |  |
| --- | --- | --- | --- | --- | --- | --- |
| **Month** | **Temp. (℃)** | **Sal.** | **Chl *a* (µg/L)** | **pH** | **DO (mg/L)** | **Quant. [µmol/(m^2^*s)]** |
|  |  |  |  |  |  |  |
| Jun 2021 | 30.94±0.02 | 32.28±0.04 | 0.63±0.10 | 8.12±0.01 | 5.66±0.11 | 321.73±22.05 |
| Jul 2021 | 29.92±0.00 | 32.30±0.00 | 1.11±0.36 | 8.15±0.00 | 6.40±0.03 | 249.76±15.57 |
| Aug 2021 | 29.90±0.00 | 32.78±0.00 | 1.15±0.17 | 8.11±0.00 | 6.07±0.01 | 70.90±3.59 |
| Sep 2021 | 29.36±0.00 | 32.91±0.00 | 0.68±0.07 | 8.10±0.00 | 6.26±0.01 | 354.33±14.78 |
| Nov 2021 | 30.09±0.00 | 32.28±0.01 | 2.80±0.55 | 8.13±0.00 | 5.74±0.03 | 139.05±13.13 |
| Dec 2021 | 27.90±0.01 | 27.73±0.01 | 1.56±0.36 | 8.08±0.00 | 5.65±0.01 | 141.45±3.43 |
| Jan 2022 | 28.48±0.00 | 31.13±0.01 | 0.80±0.17 | 8.13±0.00 | 6.37±0.02 | 32.93±0.47 |
| Mar 2022 | 27.89±0.00 | 31.58±0.01 | 2.47±0.52 | 8.05±0.00 | 5.75±0.01 | 47.30±3.53 |
| Apr 2022 | 30.72±0.00 | 31.19±0.01 | 1.67±0.71 | 8.10±0.00 | 6.13±0.01 | 149.43±12.79 |
| May 2022 | 31.77±0.00 | 31.02±0.01 | 0.85±0.07 | 8.04±0.00 | 5.06±0.03 | 44.24±1.38 |
| Jun 2022 | 30.79±0.00 | 30.97±0.00 | 0.50±0.01 | 8.18±0.00 | 6.17±0.02 | 214.64±11.06 |

Table S2. Summary the Repeated ANOVA of MQY in *P. acuta*, *P. lutea* and *T. mesenterina* in responses to low oxygen condition treatments. Significant values (*p < 0.05*) are shown in bold.

| **MQY** |  |  |  |  |  |
| --- | --- | --- | --- | --- | --- |
| **Repeated ANOVA** | **SS** | **df** | **MS** | **F** | **p** |
| ***P. acuta*** |  |  |  |  |  |
| Treatment | 0.981 | 2 | 0.491 | 214.269 | **<0.001** |
| Time | 1.414 | 1.586 | 0.892 | 241.608 | **<0.001** |
| Treatment x Time | 1.304 | 3.172 | 0.411 | 111.357 | **<0.001** |
| Error | 0.048 | 21 | 0.002 |  |  |
| ***P. lutea*** |  |  |  |  |  |
| Treatment | 0.150 | 2 | 0.075 | 15.256 | **<0.001** |
| Time | 0.437 | 3.292 | 0.133 | 20.167 | **<0.001** |
| Treatment x Time | 0.210 | 6.583 | 0.032 | 4.838 | **<0.001** |
| Error | 0.103 | 21 | 0.005 |  |  |
| ***T. mesenterina*** |  |  |  |  |  |
| Treatment | 0.294 | 2 | 0.147 | 32.855 | **<0.001** |
| Time | 0.299 | 6 | 0.050 | 19.599 | **<0.001** |
| Treatment x Time | 0.251 | 12 | 0.021 | 8.231 | **<0.001** |
| Error | 0.094 | 21 | 0.004 |  |  |

| Table S3. Summary the One-way ANOVA of Symbiodiniaceae density of P. acuta, P. lutea and T. mesenterina in responses to low oxygen condition treatments. Significant values (p < 0.05) are shown in bold. | | | | | |  |
| --- | --- | --- | --- | --- | --- | --- |
| ***Symbiodiniaceae density***  **One-way ANOVA** | **SS** | **df** | **MS** | **F** | **p** | |
| ***P. acuta*** |  |  |  |  |  | |
| Between Groups | 1.534E+13 | 3 | 5.114E+12 | 3.513 | **0.049** | |
| Within Groups | 1.747+13 | 12 | 1.456E+12 |  |  | |
| Total | 3.281+13 | 15 |  |  |  | |
| ***P. lutea*** |  |  |  |  |  | |
| Between Groups | 1.405E+12 | 3 | 4.682E+11 | 0.969 | 0.439 | |
| Within Groups | 5.797E+12 | 12 | 4.831E+11 |  |  | |
| Total | 7.201E+12 | 15 |  |  |  | |
| ***T. mesenterina*** |  |  |  |  |  | |
| Between Groups | 2.064E+12 | 3 | 6.881E+11 | 1.618 | 0.237 | |
| Within Groups | 5.104E+12 | 12 | 4.253E+11 |  |  | |
| Total | 7.168E+12 | 15 |  |  |  | |

Table S4. Summary the One-way ANOVA of chlorophyll *a* concentration of *P. acuta*, *P. lutea* and *T. mesenterina* in responses to low oxygen condition treatments. Significant values (*p < 0.05*) are shown in bold.

| **Chlorophyll a concentration**  **One-way ANOVA** | **SS** | **df** | **MS** | **F** | **p** |
| --- | --- | --- | --- | --- | --- |
| ***P. acuta*** |  |  |  |  |  |
| Between Groups | 26.845 | 3 | 8.948 | 2.525 | 0.107 |
| Within Groups | 42.529 | 12 | 3.544 |  |  |
| Total | 69.374 | 15 |  |  |  |
| ***P. lutea*** |  |  |  |  |  |
| Between Groups | 0.552 | 3 | 0.184 | 0.022 | 0.995 |
| Within Groups | 98.996 | 12 | 8.250 |  |  |
| Total | 99.548 | 15 |  |  |  |
| ***T. mesenterina*** |  |  |  |  |  |
| Between Groups | 61.522 | 3 | 20.507 | 5.433 | **0.014** |
| Within Groups | 45.292 | 12 | 3.774 |  |  |
| Total | 106.813 | 15 |  |  |  |

Table S5. Summary the One-way ANOVA of chlorophyll *c_2_* concentration of *P. acuta*, *P. lutea* and *T. mesenterina* in responses to low oxygen condition treatments. Significant values (*p < 0.05*) are shown in bold.

| **Chlorophyll c_2_ concentration**  **One-way ANOVA** | **SS** | **df** | **MS** | **F** | **p** |
| --- | --- | --- | --- | --- | --- |
| ***P. acuta*** |  |  |  |  |  |
| Between Groups | 1.684 | 3 | 0.561 | 1.421 | 0.285 |
| Within Groups | 4.740 | 12 | 0.395 |  |  |
| Total | 6.424 | 15 |  |  |  |
| ***P. lutea*** |  |  |  |  |  |
| Between Groups | 1.834 | 3 | 0.611 | 0.442 | 0.727 |
| Within Groups | 16.598 | 12 | 1.383 |  |  |
| Total | 18.432 | 15 |  |  |  |
| ***T. mesenterina*** |  |  |  |  |  |
| Between Groups | 2.570 | 3 | 0.857 | 1.998 | 0.168 |
| Within Groups | 5.147 | 12 | 0.429 |  |  |
| Total | 7.717 | 15 |  |  |  |

Table S6. Summary of ANOVAs of respiration rate of *P. acuta*, *P. lutea* and *T. mesenterina* in responses to low oxygen condition treatments. Significant values (*p < 0.05*) are shown in bold.

| **Respiration rate** | **SS** | **df** | **MS** | **F** | **p** |
| --- | --- | --- | --- | --- | --- |
| One-way ANOVA  ***P. acuta*** |  |  |  |  |  |
| Between Groups | 1185.728 | 2 | 592.864 | 223.264 | **<0.001** |
| Within Groups | 55.764 | 21 | 2.655 |  |  |
| Total | 1241.492 | 23 |  |  |  |
| Repeated ANOVA  ***P. lutea*** |  |  |  |  |  |
| Treatments | 1862.277 | 2 | 931.139 | 142.316 | **<0.001** |
| Days | 1.149 | 2 | 0.5744 | 0.761 | 0.478 |
| Treatments * Days | 8.818 | 4 | 2.204 | 2.919 | **0.042** |
| Error | 18.127 | 24 | 0.755 |  |  |
| Repeated ANOVA  ***T. mesenterina*** |  |  |  |  |  |
| Treatments | 594.177 | 2 | 297.089 | 357.496 | **<0.001** |
| Days | 5.164 | 2 | 2.582 | 8.832 | **0.003** |
| Treatments * Days | 6.270 | 4 | 1.567 | 5.361 | **.008** |
| Error | 4.093 | 14 | 0.292 |  |  |

Table S7. Summary of ANOVAs of net primary production rate of *P. acuta*, *P. lutea* and *T. mesenterina* in responses to low oxygen condition treatments. Significant values (*p < 0.05*) are shown in bold.

| **Net primary production** | **SS** | **df** | **MS** | **F** | **p** |
| --- | --- | --- | --- | --- | --- |
| One-way ANOVA  ***P. acuta*** |  |  |  |  |  |
| Between Groups | 114.981 | 2 | 57.490 | 1.602 | 0.225 |
| Within Groups | 753.423 | 21 | 35.877 |  |  |
| Total | 868.403 | 23 |  |  |  |
| Repeated ANOVA  ***P. lutea*** |  |  |  |  |  |
| Treatments | 905.506 | 2 | 452.753 | 24.673 | **<0.001** |
| Days | 8.924 | 2 | 4.462 | 0.952 | 0.400 |
| Treatments * Days | 14.522 | 4 | 3.631 | 0.774 | 0.553 |
| Error | 112.531 | 24 | 4.689 |  |  |
| Repeated ANOVA  ***T. mesenterina*** |  |  |  |  |  |
| Treatments | 589.832 | 2 | 294.916 | 253.947 | **<0.001** |
| Days | 83.196 | 2 | 41.598 | 19.412 | **<0.001** |
| Treatments * Days | 79.855 | 4 | 19.964 | 9.316 | **<0.001** |
| Error | 51.430 | 24 | 2.143 |  |  |
|  |  |  |  |  |  |

Table S8. Summary of ANOVAs of gross primary production rate of *P. acuta*, *P. lutea* and *T. mesenterina* in responses to low oxygen condition treatments. Significant values (*p < 0.05*) are shown in bold.

| **Gross primary production** | **SS** | **df** | **MS** | **F** | **p** |
| --- | --- | --- | --- | --- | --- |
| One-way ANOVA  ***P. acuta*** |  |  |  |  |  |
| Between Groups | 563.052 | 2 | 281.526 | 6.296 | **0.007** |
| Within Groups | 938.944 | 21 | 44.712 |  |  |
| Total | 1501.996 | 23 |  |  |  |
| Repeated ANOVA  ***P. lutea*** |  |  |  |  |  |
| Treatments | 289.636 | 2 | 144.818 | 10.701 | **0.002** |
| Days | 5.507 | 2 | 2.753 | 0.408 | 0.669 |
| Treatments * Days | 38.788 | 4 | 9.697 | 1.439 | 0.249 |
| Error | 175.245 | 26 | 6.740 |  |  |
| Repeated ANOVA  ***T. mesenterina*** |  |  |  |  |  |
| Treatments | 29.078 | 2 | 14.539 | 3.705 | .059 |
| Days | 156.386 | 2 | 78.193 | 21.584 | **<0.001** |
| Treatments * Days | 28.615 | 4 | 7.154 | 1.975 | 0.134 |
| Error | 79.700 | 22 | 3.623 |  |  |
|  |  |  |  |  |  |

Table S9. Summary of ANOVAs of calcification rate of *P. acuta*, *P. lutea* and *T. mesenterina* in responses to low oxygen condition treatments. Significant values (*p < 0.05*) are shown in bold.

| **Calcification rate** | **SS** | **df** | **MS** | **F** | **p** |
| --- | --- | --- | --- | --- | --- |
| One-way ANOVA  ***P. acuta*** |  |  |  |  |  |
| Between Groups | 78.811 | 2 | 39.406 | 6.629 | **0.009** |
| Within Groups | 89.164 | 15 | 5.944 |  |  |
| Total | 167.975 | 17 |  |  |  |
| Repeated ANOVA  ***P. lutea*** |  |  |  |  |  |
| Treatments | 451.555 | 2 | 225.778 | 25.804 | **<0.001** |
| Days | 0.014 | 2 | 0.007 | 0.002 | 0.998 |
| Treatments * Days | 12.977 | 4 | 3.244 | 0.909 | 0.476 |
| Error | 78.548 | 22 | 3.570 |  |  |
| Repeated ANOVA  ***T. mesenterina*** |  |  |  |  |  |
| Treatments | 55.735 | 2 | 27.867 | 21.867 | **0.001** |
| Days | 47.339 | 2 | 23.669 | 24.247 | **<0.001** |
| Treatments * Days | 42.466 | 4 | 10.616 | 10.876 | **<0.001** |
| Error | 15.619 | 16 | 0.976 |  |  |

Table S10. Summary of One-Way ANOVA of ambient treatment of *P. acuta*, *P. lutea* and *T. mesenterina* in times. Significant values (*p < 0.05*) are shown in bold.

| **MQY** | **SS** | | **df** | | **MS** | | **F** | | **p** | |  |
| --- | --- | --- | --- | --- | --- | --- | --- | --- | --- | --- | --- |
| One-way ANOVA  ***P. acuta*** |  | |  | |  | |  | |  | |  |
| Between Groups | .000 | | 2 | | .000 | | .110 | | 0.897 | |  |
| Within Groups | .007 | | 21 | | .000 | |  | |  | |  |
| Total | .007 | | 23 | |  | |  | |  | |  |
| One-way ANOVA  ***P. lutea*** | |  | |  | |  | |  | |  | |
| Between Groups | | .018 | | 6 | | .003 | | 1.716 | | 0.137 | |
| Within Groups | | .088 | | 49 | | .002 | |  | |  | |
| Total | | .106 | | 55 | |  | |  | |  | |
| One-way ANOVA  ***T. mesenterina*** | |  | |  | |  | |  | |  | |
| Between Groups | | .009 | | 6 | | .002 | | .493 | | 0.810 | |
| Within Groups | | .156 | | 49 | | .003 | |  | |  | |
| Total | | .166 | | 55 | |  | |  | |  | |
|  | |  | |  | |  | |  | |  | |

Figure S1. Records of Temperature in Kham Island throughout the period from June 2021 to June 2022.


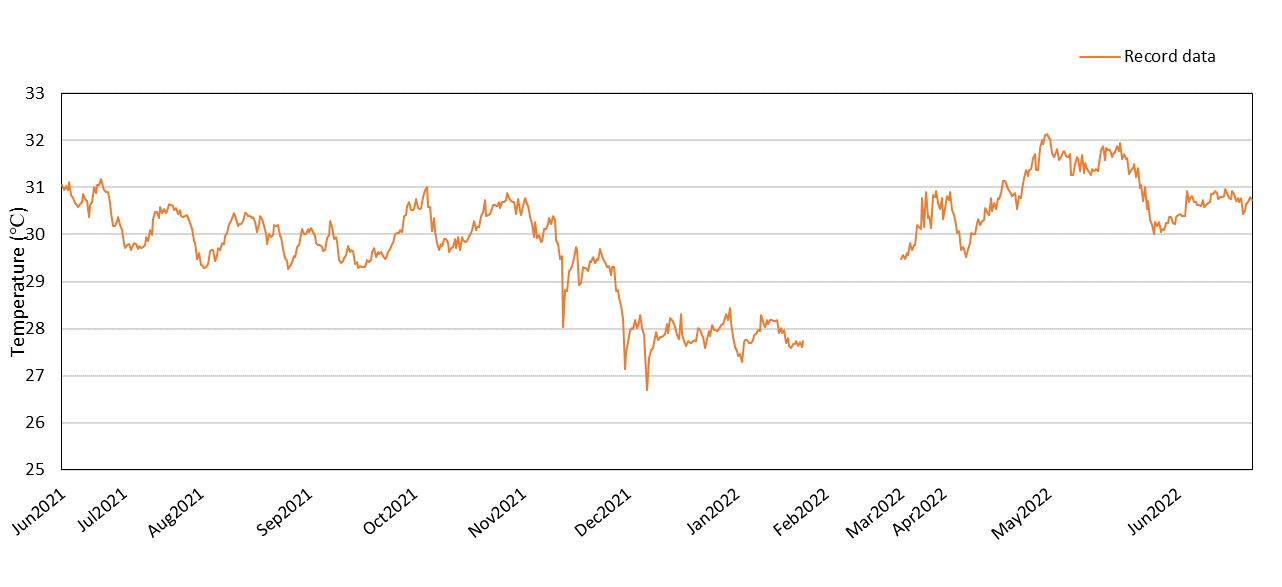

Supplement: Supplemental Information 1 [file peerj-12-18095-s001.docx]
